# The incremental value of interatrial septum motion in predicting thrombus or spontaneous echo contrast in patients with non-valvular atrial fibrillation: an observational study on transesophageal echocardiography

**DOI:** 10.3389/fcvm.2024.1366180

**Published:** 2024-10-16

**Authors:** Decai Zeng, Shuai Chang, Xiaofeng Zhang, Yanfeng Zhong, Cai Yongzhi, Tongtong Huang, Ji Wu

**Affiliations:** Department of Ultrasonic Medicine, The First Affiliated Hospital of Guangxi Medical University, Nanning, China

**Keywords:** atrial fibrillation, interatrial septum motion, transesophageal echocardiography, spontaneous echo contrast, thrombus

## Abstract

**Background:**

The thickness and motion of the Interatrial Septum (IAS) possibly serves as indicators of both structural and functional remodeling of left atrium. This study aims to use transesophageal echocardiography (TEE) to assess IAS motion in non-valvular atrial fibrillation (NVAF) and investigate its correlation with the risk of spontaneous echo contrast (SEC) and thrombus (TH).

**Methods:**

We conducted a cross-sectional study on 318 patients with NVAF who underwent transthoracic echocardiography and TEE. IAS motion was defined as the maximum displacement of IAS observed throughout the cardiac cycles using M-mode TEE.

**Results:**

The prevalence of SEC/TH was 39.9% (127/318) in the overall group. In paroxysmal atrial fibrillation (PAF), the prevalence rate of SEC/TH was 25.3% (47/186), compared to 60.6% (80/132) in persistent atrial fibrillation (PeAF). Multivariable analyses showed that LA volume index (LAVI), mean E/e’, S/D ratio, IAS motion and CHA2DS2-VASc scores were significantly associated with SEC/TH. Patients with lower IAS motion showed a higher prevalence of SEC/TH compared to those with higher IAS motion (71.3% vs. 11.6%; *P* < 0.001). The IAS motion under sinus rhythm could better predict risk of SEC/TH, while the predictive efficacy under AF rhythm was slightly attenuated but still had a high AUC value (0.779). A significant positive correlation was observed between the IAS motion and the LAA filling velocity (PAF *r* = 0.47; *P*<0.001 and PeAF *r* = 0.38; *P* < 0.001, respectively), LAA emptying velocity (PAF *r* = 0.55; *P* < 0.001 and PeAF *r* = 0.47; *P* < 0.001, respectively) and LAVI (PAF *r* = 0.59; *P* < 0.001 and PeAF *r* = 0.44; *P* < 0.001, respectively). The integration of the IAS motion to the CHA2DS2-VASc, LAVI and mean E/e’ provided important incremental predictive value of SEC/TH (AUC = 0.859 vs. 0.826, *P* = 0.02).

**Conclusion:**

IAS motion measured by TEE correlates well with LAA flow velocity and LA size and is independently associated with SEC/TH in NVAF. Additionally, lower IAS motion is associated with a higher prevalence of SEC/TH. Furthermore, the integration of IAS motion to CHA2DS2-VASc, LAVI and mean E/e’ can provide additional value for the prediction of SEC/TH.

## Introduction

Atrial fibrillation (AF) is the most common cardiac arrhythmia, and its incidence is increasing due to the growing elderly population (1). It results in the loss of coordinated myocardial contraction, leading to a rapid and irregular heartbeat. This disruption hampers the atria's ability to contract effectively, impairing their normal function. AF causes abnormal left atrial (LA) hemodynamics, which subsequently increases the risk of stroke, heart failure, and other complications related to the heart (2).

The interatrial septum (IAS) is conventionally considered a fibromuscular structure that separates the right atrium from the left atrium (LA). Although it does not directly trigger atrial fibrillation, The structure and function of the interatrial septum (IAS) could potentially affect atrial fibrillation (AF) by impacting atrial electromechanical function and interacting with neighboring structures that are essential in the initiation and maintenance of AF (3, 4). Previous studies have suggested significant correlations between thickness of the IAS and parameters associated with atrial structural and functional remodeling in patients with AF (5, 6). Nonetheless, the utilization of IAS motion in the context of AF remains somewhat restricted. As well known, the normal motion of the interatrial septum is a crucial component of the synchronized contraction and relaxation of the atria. The shape and movement of the interatrial septum have also been found to be closely related to LA pressure (7). Fibrotic remodeling of the IAS can occur in the presence of AF and contribute to abnormal electrical conduction, it may also impact the regular movement of the interatrial septum (2). Assessing the motion of the interatrial septum has the potential to indicate atrial pressures and function, thereby offering valuable insights into the underlying pathophysiological mechanisms involved in AF. Besides, the evaluation of IAS motion might have implications for prognosis and could guide decisions on managing and treating AF. A deeper understanding of the role of the IAS motion in the pathophysiology of AF has the potential to enhance stroke risk assessment.

Over the past two decades, there has been substantial technological advancement in advanced imaging technologies such as cardiac magnetic resonance (CMR), transthoracic and transesophageal echocardiography (TEE), and computed tomography. TEE is likely the most effective imaging modality for describing the movement of IAS (8). The presence of LA/Left atrial appendage (LAA) thrombus (TH) and spontaneous echo contrast (SEC) is frequently observed during TEE examination in patients with non-valvular atrial fibrillation (NVAF), which lead to a higher risk of embolism and worse clinical outcomes in stroke patients.

Therefore, this study aims to utilize TEE to evaluate the motion of the atrial septum in patients with NVAF and investigate its correlation with the risk of SEC/TH. Further, we explored the added value of including IAS motion in prediction stroke risk of NVAF.

## Methods

**Figure 1:**
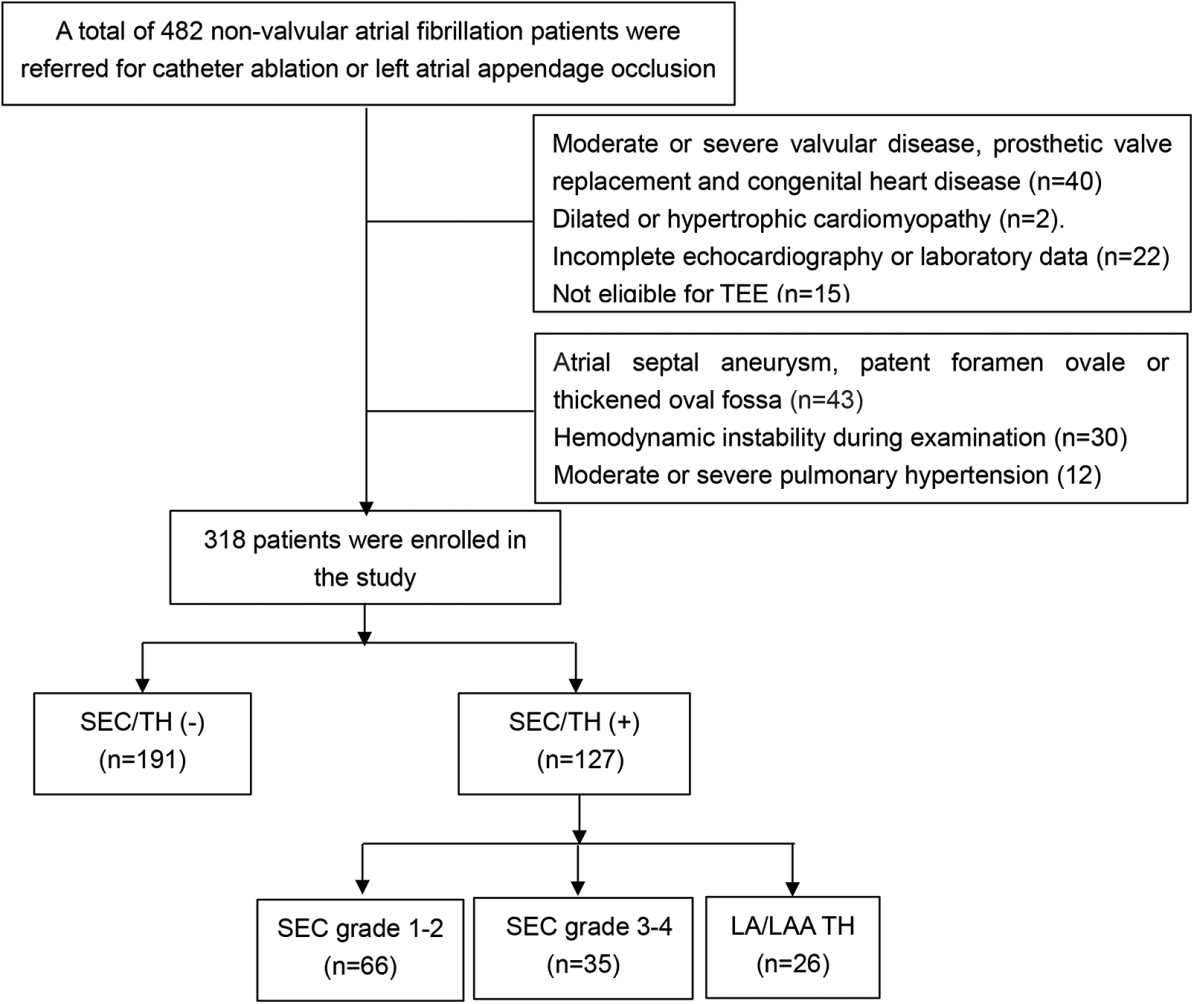
Flow chart of the screening process for the selection of eligible participants (LA, left atria; LAA, left atrial appendage; SEC, spontaneous echo contrast; TEE, transesophageal echocardiography; TH, thrombosis).

This study screened all eligible patients with non-valvular atrial fibrillation who were considered for radiofrequency ablation and/or LAA occlusion at the First Affiliated Hospital of Guangxi Medical University from January 2020 to April 2023. The inclusion criteria consisted of patients with either paroxysmal or persistent atrial fibrillation (PeAF). Paroxysmal atrial fibrillation (PAF) was defined as a spontaneous return to sinus rhythm within 7 days, while PeAF referred to AF that lasted longer than 7 days and required medication or electric shock for conversion to sinus rhythm. The exclusion criteria were as follows: (1) Moderate or severe valvular disease, prosthetic valve replacement and congenital heart disease (2) Dilated or hypertrophic cardiomyopathy, (3) Incomplete echocardiography or laboratory data. (4) Not eligible for TEE; (5) atrial septal aneurysm, patent foramen ovale or thickened oval fossa; (6). Hemodynamic instability during TEE examination. (7) Moderate or severe pulmonary hypertension. The flowchart of the selection of study participants was shown in Figure 1. The final analysis included a total of 318 patients with NVAF. This study was approved by the Ethics Committee of the First Affiliated Hospital of Guangxi Medical University, and all patients provided their informed consent by signing the appropriate form. Demographic and clinical information was obtained by retrospectively analyzing electronic medical records.

The basic information of patients, such as height, weight, and blood pressure, was routinely collected. Hypertension was defined as having a systolic blood pressure equal to or exceeding 140 mm Hg, or a diastolic blood pressure equal to or exceeding 90 mm Hg, or being on antihypertensive medication. Diabetes was defined as having a fasting blood glucose level of 126 mg/dl or higher, or currently using insulin or hypoglycemic agents. Dyslipidemia was defined as having a total serum cholesterol level of more than 240 mg/dl, or using lipid-lowering drugs. The history of diseases included hypertension, diabetes mellitus, coronary heart disease, and previous stroke were obtained. To evaluate the risk of stroke, The CHA_2_DS_2_-Vasc score was determined based on the relevant factors. Fasting blood specimens were collected and biochemically examined for the concentration of fasting plasma glucose, NT-proBNP, troponin I, Scr, and Ccr upon admission.

All patients included in the study underwent echocardiography before radiofrequency ablation and/or LAA occlusion. The TEE and TTE procedures were performed using the Philips EPIQ 7C ultrasound imaging system from Philips Medical Systems (Bothell, WA, USA). For TTE examination, the 2D parameters are routinely measured using S5-1 probe with a frequency range of 1–5 MHz for the three-dimensional echocardiography, the real-time X5-1 probe was utilized. All the parameters were calculated by averaging three consecutive cardiac cycles during Sinus rhythm, while measurements obtained from echocardiography were averaged over five consecutive cardiac cycles during AF.

The patient was positioned on their left side and connected to an ECG machine. Six cardiac cycles were conducted to collect and store images. Measurements were taken for various parameters, including the left ventricular end-diastolic diameter (LVEDD), left ventricular end-systolic diameter (LVESD), septal wall thickness (SWT), and left ventricular posterior wall thickness (PWT). The diameter of the LA was obtained using two-dimensional echocardiography from the long axis view of the left ventricle. The mitral flow spectrum was obtained using Pulse Doppler from the apical four-chamber section to measure the E peak. Tissue Doppler was used to measure e’ on the septal side and e’ on the lateral side. The E/e’ ratio for the septal side and lateral side were calculated separately, and the mean E/e’ was obtained. A transthoracic three-dimensional matrix probe was used to collect images in the standard apical four-chamber view. The Heart Model mode was used to obtain three-dimensional volume data of the left heart, including the left ventricular end-diastolic volume (LVEDV), left ventricular end-systolic volume (LVESV), ejection fraction (EF), and LA maximum volume (LAV max). The left ventricular mass can be calculated using the formula: LV mass = 0.8{1.04[(SWT + LVEDD + PWT)3—LVEDD^3^]} + 0.6, where SWT represents the left ventricular end-diastolic septal wall thickness, LVEDD represents the left ventricular end-diastolic diameter, and PWT represents the left ventricular end-diastolic posterior wall thickness (9). The LA volume index (LAVI) and left ventricular mass index (LVMI) were calculated based on the patient's body surface area.

After contraindications of TEE were excluded, the patient signed the informed consent and was instructed to use tetracaine hydrochloride for throat anesthesia after fasting for more than 8 h. The probe was placed at the level of the middle segment of the esophagus with the patient in the left lateral decubitus position. A comprehensive examination of the LA and LAA was conducted to identify any presence of thrombus and/or SEC.

**Figure 2:**
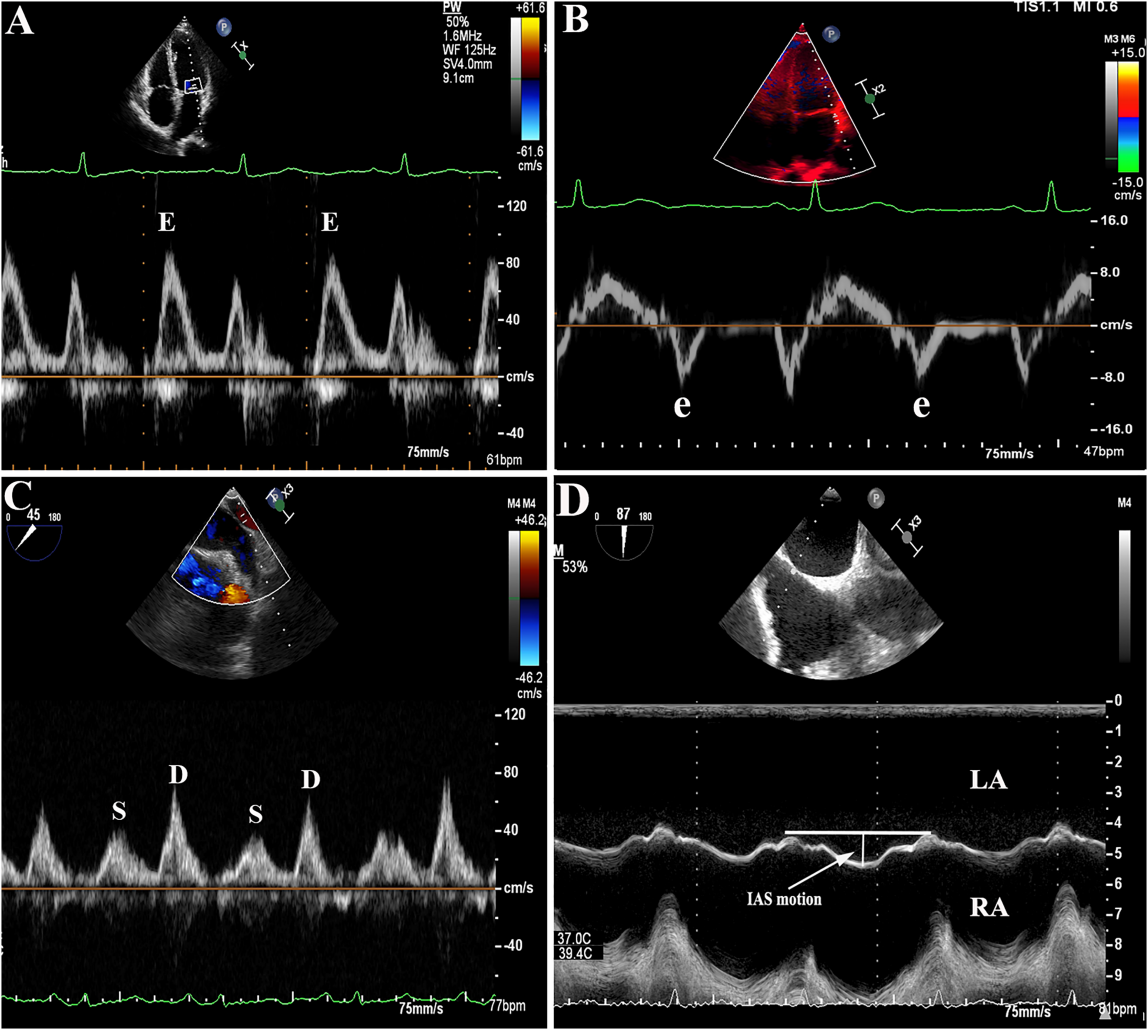
**(A–B)**: Representative images of E/e' ratio measurement by transthoracic echocardiography (TTE). **(C)**: Example figures of S/D ratio, **(D)**: interatrial septal (IAS) motion measurement by transesophageal echocardiography (TEE).

Pulse Doppler was used for measuring the peak velocity of blood flow during filling and emptying of the LAA. The sampling line was placed in the middle of the LAA orifice on the 45° section. Similarly, on the 45° section, the sampling line was positioned 1 cm inside the left pulmonary vein orifice to ensure that the sound beam was parallel to the direction of blood flow in the pulmonary vein. The spectrum of the pulmonary vein was obtained, and the S and D peaks were measured to determine the S/D ratio. The movement of the IAS was monitored through a two-dimensional and M-mode system at the mid-esophageal aortic valve short-axis view (90°). To obtain the M-mode echocardiography of the IAS, a cursor was inserted through the thin part (oval fossa) of the IAS in the cross-sectional image at a perpendicular angle. The IAS motion was defined as the maximum IAS displacement observed throughout the cardiac cycles (as shown in Figure 2). All echocardiographic parameters including IAS motion were continuously obtained for 5 cardiac cycles and averaged during AF rhythm, and for 3 cardiac cycles during sinus rhythm.

SEC is defined as a smoke-like echo with swirling blood flow in the LA and/or LAA. A thrombus is a blood clot that forms in the LA, typically in the LAA in AF patients.

Continuous variables were compared using mean ± SD or median with interquartile range, differences were assessed for statistical significance using either the Student *t*-test or Mann–Whitney *U* test based on the distribution. Categorical variables were presented as frequencies and percentage, and were compared using the *χ*^2^ test or Fisher exact test. Baseline characteristics, including laboratory parameters and echocardiographic parameters, were compared between patients with and without SEC/TH. Univariable and multivariable logistic regression analyses were conducted to identify the factors influencing SEC/TH. Factors associated with a *P* < 0.05 level were included in the multivariable analysis. Pearson's correlation was applied for the purpose of conducting correlation analysis. Receiver operating characteristic curves were used to determine the sensitivity and specificity of the cut-off point for predicting SEC/TH. Statistical analyses were performed using R (version 3.2.3, R Project for Statistical Computing).

## Results

In this study, 318 cases were ultimately included. Table 1 summarizes the baseline characteristics of all subjects who were enrolled. The mean age was 59.5 ± 10.7 years, with 93 subjects (29%) being female. Out of all subjects, 132 (42%) had PAF and 186 (58%) had PeAF. The CHA_2_DS_2_-VASc score had a median of 2(IQR, 1–4). The prevalence of SEC/TH was 39.9% (127/318) in the overall group. The prevalence rate of SEC/TH was 25.3% (47/186) in PAF, compared to 60.6% (80/132) in PeAF.

**Table 1:** Baseline Characteristics of the 354 patients stratified by the presence or absence of SEC/TH.

Variable	Overall (*n* = 318)	No SEC/TH (*n* = 191)	SEC/TH (*n* = 127)	*P* value
Age, years	59.48 ± 10.68	57.53 ± 11.56	62.41 ± 8.44	<0.001
Female sex, *n* (%)	93 (29)	61 (32)	32 (25)	0.243
Body surface area, m²	1.72 ± 0.2	1.71 ± 0.18	1.74 ± 0.22	0.253
Systolic blood pressure, mmHg	128.18 ± 18.94	128.45 ± 19.04	127.78 ± 18.85	0.757
Diastolic blood pressure, mmHg	79.76 ± 11.92	78.84 ± 11.24	81.14 ± 12.79	0.101
Medical history				
Hypertension, *n* (%)	161 (51)	84 (44)	77 (61)	0.005
Diabetes, *n* (%)	42 (13)	19 (10)	23 (18)	0.053
Dyslipidemia, *n* (%)	99 (31)	67 (35)	32 (25)	0.082
Vascular disease, *n* (%)	133 (42)	73 (38)	60 (47)	0.138
Coronary heart disease, *n* (%)	61 (19)	31 (16)	30 (24)	0.135
History of heart failure, *n* (%)	20 (6)	5 (2)	15 (11)	0.001
Persistent AF, *n* (%)	132 (42)	52 (27)	80 (63)	<0.001
CHA_2_DS_2_ -VASc score	2 (1,4)	2 (1,3)	3 (2,4)	<0.001
Laboratory data				
Scr, µmol/L	84.19 ± 29.63	81.41 ± 20.06	88.36 ± 39.65	0.07
Ccr, ml/min	78.09 ± 18.07	81.85 ± 17.73	72.44 ± 17.15	<0.001
NT-proBNP, pg/ml	574 (145, 1065)	281 (83, 948)	960 (513, 1982)	<0.001
Troponin I, ng/L	4 (2, 9.75)	3 (2, 7)	7 (4, 14)	<0.001
Medication				
Antiplatelet, *n* (%)	35 (11)	19 (10)	16 (13)	0.578
Warfarin, *n* (%)	19 (6)	5 (3)	14 (11)	0.004
NOAC, *n* (%)	205 (64)	120 (63)	85 (67)	0.529
Echocardiographic parameters				
LVEDV, ml	126.52 ± 36.13	121.32 ± 34.64	134.33 ± 37.04	0.002
LVESV, ml	49.85 ± 30.11	45.21 ± 28.86	56.83 ± 30.71	<0.001
LVMI, g/m^2^	132.01 ± 37.2	124.55 ± 35.25	143.22 ± 37.36	<0.001
LV ejection fraction,%	62.88 ± 10.96	65.22 ± 9.41	59.36 ± 12.17	<0.001
LAVI, ml/m^2^	41.97 ± 17.32	35.16 ± 14.89	52.21 ± 15.63	<0.001
Mean E/e’	9.06 ± 3.45	8.34 ± 3	10.16 ± 3.8	<0.001
S/D ratio	1.03 ± 0.56	1.22 ± 0.58	0.75 ± 0.38	<0.001
IAS motion (mm)	7.36 ± 2.77	8.55 ± 2.67	5.58 ± 1.77	<0.001

Values are mean ± SD, *n* (percentage), or median (25th, 75th percentile).

AF, atrial fibrillation; LAVI, left atrial volume index; LV, left ventricle; LVEDV, left ventricular end diastolic volume; LVESV, left ventricular end systolic volume; LVMI, left ventricular mass index; IAS, interatrial septum, SEC/TH, spontaneous echo contrast/thrombus.

**Figure 3:**
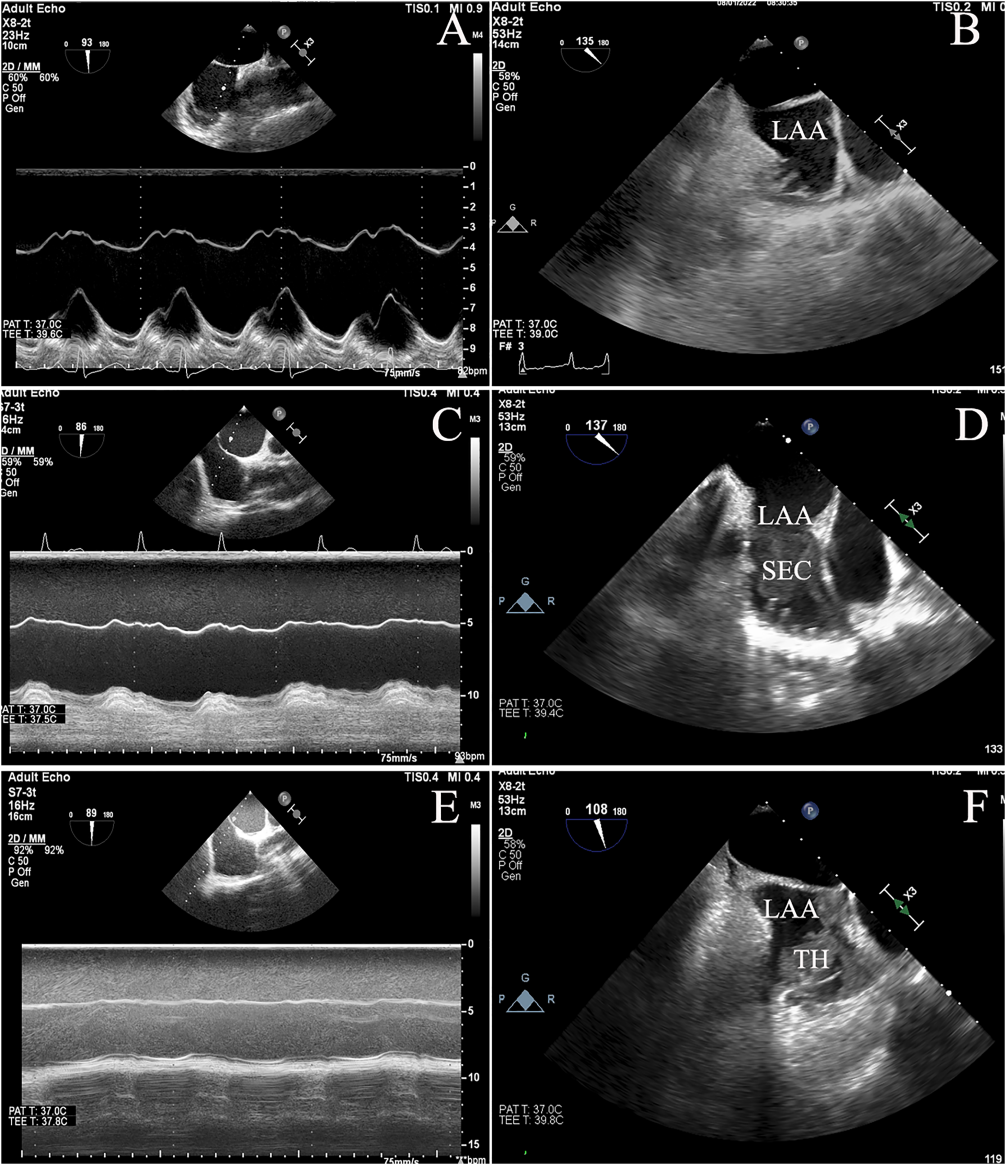
The figure depicted the varying degrees of interatrial septal (IAS) motion, ranging from normal **(A)** to reduced **(C)** or restricted **(E)** movement, and showed the correlation between IAS motion and spontaneous echo contrast / thrombosis (SEC/TH). **(B)**: No SEC/TH; **(D)**: SEC; **(F)**: thrombus.

There was no difference in sex, body surface area, systolic blood pressure, and diastolic blood pressure between patients with SEC/TH compared to those without SEC/TH. However, a significant statistical difference was found between the two groups in Hypertension, persistent AF, and heart failure. Although there was no significant difference in the prevalence of diabetes, dyslipidemia, vascular disease, and coronary artery disease, the SEC/TH group had a significantly higher CHA_2_DS_2_-VASc scores than the group without SEC/TH. In terms of laboratory measurements, the SEC/TH group showed significantly decreased Ccr, higher NT-proBNP levels, and elevated Troponin I levels compared to the group without SEC/TH (all *P* < 0.05). There was a higher usage of Warfarin in patients with SEC/TH (*P* < 0.05). Table 1 also summarizes the echocardiographic parameters of the study population. It was found that LVEDV, LVESV, LV mass index, LAVI, and Mean E/e’ were higher in patients with SEC/TH compared to those without SEC/TH (all *P* < 0.05). Moreover, the LV ejection fraction, S/D ratio, and IAS motion in the SEC/TH (+) group were lower than those in the SEC/TH (−) group (all *P* < 0.05). Figure 3 displays the correlation between IAS motion and the occurrence of SEC/TH. The SEC/TH (+) group was further subdivided into three subgroups, but subgroup analyses showed no differences in IAS motion among subgroups (Supplementary Figure S1).

Age, hypertension, diabetes, persistent AF, heart failure, CHA_2_DS_2_-VASc scores, Ccr, NT-proBNP, Warfarin, and choreographic parameters were found to have a significant association with SEC/TH in the univariable logistic regression analysis (all *P* < 0.05; Table 2).

**Table 2:** Univariate and multivariate logistic regression analysis of LAT/SEC presence based on peri-procedural data.

Variable	Univariate analysis		Multivariate analysis	
	OR (95% CI)	*P*-value	OR (95% CI)	*P*-value
Age, years	1.048 (1.024–1.073)	<0.001	0.972 (0.930–1.015)	0.204
Female sex, *n* (%)	0.718 (0.431–1.181)	0.196		
Body surface area, m²	2.008 (0.635–6.433)	0.236		
Systolic blood pressure, mmHg	0.998 (0.986–1.01)	0.757		
Diastolic blood pressure, mmHg	1.016 (0.997–1.036)	0.093		
Medical history				
Hypertension, *n* (%)	1.962 (1.246–3.109)	0.004	0.863 (0.387–1.887)	0.716
Diabetes, *n* (%)	2.002 (1.042–3.888)	0.038	1.365 (0.514–3.691)	0.533
Dyslipidemia, *n* (%)	0.623 (0.375–1.021)	0.063		
Vascular disease, *n* (%)	1.448 (0.919–2.283)	0.111		
Coronary artery disease, *n* (%)	1.596 (0.908–2.805)	0.103		
History of heart failure, *n* (%)	4.242 (1.555–13.50)	0.007	2.209 (0.483–12.13)	0.327
Persistent AF, *n* (%)	4.55 (2.83–7.413)	<0.001	1.832 (0.951–3.525)	0.069
CHA_2_DS_2_-VASc scores	1.488 (1.282–1.742)	<0.001	1.469 (1.102–1.988)	0.010
Laboratory data				
Scr, µmol/L	1.01 (1.001–1.021)	0.060		
Ccr, ml/min	0.969 (0.955–0.982)	<0.001	0.985 (0.965–1.003)	0.121
NT-proBNP, ng/ml	2.076 (1.601–2.793)	<0.001	1.058 (0.782–1.463)	0.719
Troponin I, ng/L	1.015 (1.003–1.03)	0.025	0.991 (0.977–1.005)	0.217
Medication				
Antiplatelet, *n* (%)	1.305 (0.637–2.644)	0.460		
Warfarin, *n* (%)	4.609 (1.713–14.57)	0.004	0.960 (0.248–4.186)	0.955
NOAC, *n* (%)	1.197 (0.749–1.928)	0.454		
Echocardiographic parameters				
LVEDV, ml	1.01 (1.004–1.017)	0.003	1.021 (0.997–1.047)	0.084
LVESV, ml	1.014 (1.006–1.023)	0.001	0.966 (0.929–1.002)	0.076
LVMI, g/m^2^	1.014 (1.008–1.021)	<0.001	0.988 (0.974–1.002)	0.104
LV ejection fraction,%	0.95 (0.928–0.971)	<0.001	0.955 (0.899–1.010)	0.119
LAVI, ml/m^2^	1.073 (1.054–1.094)	<0.001	1.029 (1.004–1.057)	0.025
Mean E/e’	1.171 (1.093–1.259)	<0.001	1.113 (1.003–1.240)	0.047
S/D ratio	0.121 (0.065–0.213)	<0.001	0.271 (0.127–0.556)	0.001
IAS motion (mm)	0.557 (0.479–0.637)	<0.001	0.718 (0.597–0.853)	<0.001

AF, atrial fibrillation; LAVI, left atrial volume index; LV, left ventricle; LVEDV, left ventricular end diastolic volume; LVESV, left ventricular end systolic volume; LVMI, left ventricular mass index; IAS, interatrial septum; SEC/TH, spontaneous echo contrast/thrombus.

To further identify independent risk factors for SEC/TH, multivariable analyses were conducted. These analyses revealed that LA parameters, such as LAVI, mean E/e’, S/D ratio, and IAS motion, were significantly associated with SEC/TH. Furthermore, CHA_2_DS_2_-VASc scores were also independently associated with SEC/TH.

**Figure 4:**
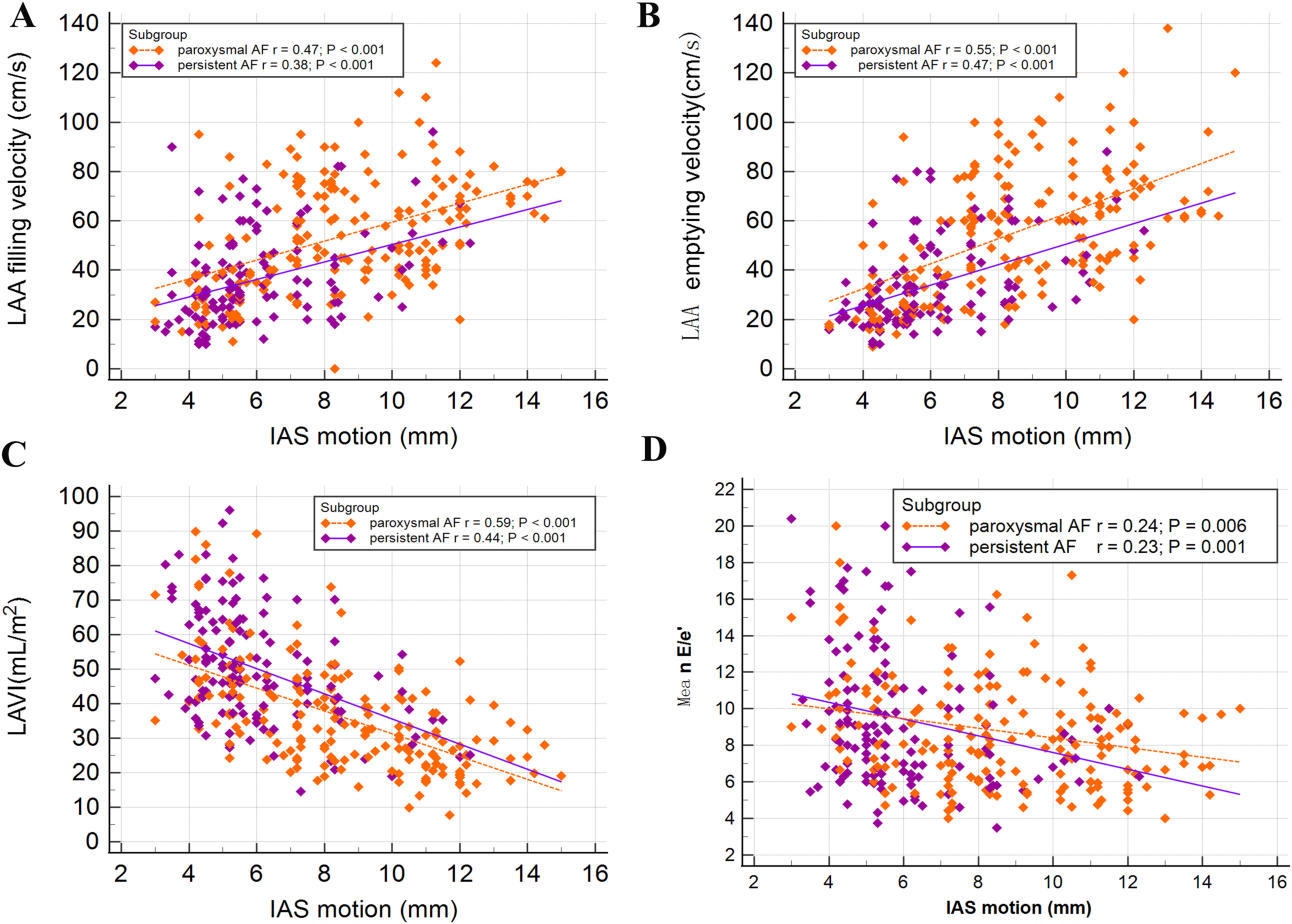
Associations between interatrial septal (IAS) motion and other echocardiographic parameters were examined in patients with paroxysmal atrial fibrillation (PAF) or persistent atrial fibrillation (PeAF). A significant positive relationship was observed between IAS motion and left atrial appendage (LAA) filling velocity **(A)**, as well as emptying velocity **(B)**. On the other hand, a negative correlation was found between IAS motion and LAVI **(C)**. Furthermore, a very weak correlation was identified between IAS motion and mean E/e’ **(D)**.

Figure 4 showed the Pearson correlation coefficients, stratified by PeAF and PAF, indicating the relationship between the IAS motion and LAA hemodynamic parameters, as well as LAVI and mean E/e’. The results indicated a significant positive correlation between the IAS motion and the filling speed of LAA (PAF *r* = 0.47; *P* < 0.001 and PeAF *r* = 0.38; *P* < 0.001, respectively), as well as the emptying speed of LAA (PAF *r* = 0.55; *P* < 0.001 and PeAF *r* = 0.47; *P* < 0.001, respectively). Additionally, there was a significant negative correlation with the LAVI (PAF *r* = 0.59; *P* < 0.001 and PeAF *r* = 0.44; *P* < 0.001, respectively). However, we identified a poor correlation between the IAS motion and mean E/e’ both in patients with PeAF (*r* = 0.23; *P* = 0.001) and PAF (*r* = 0.24; *P* = 0.006).

**Figure 5:**
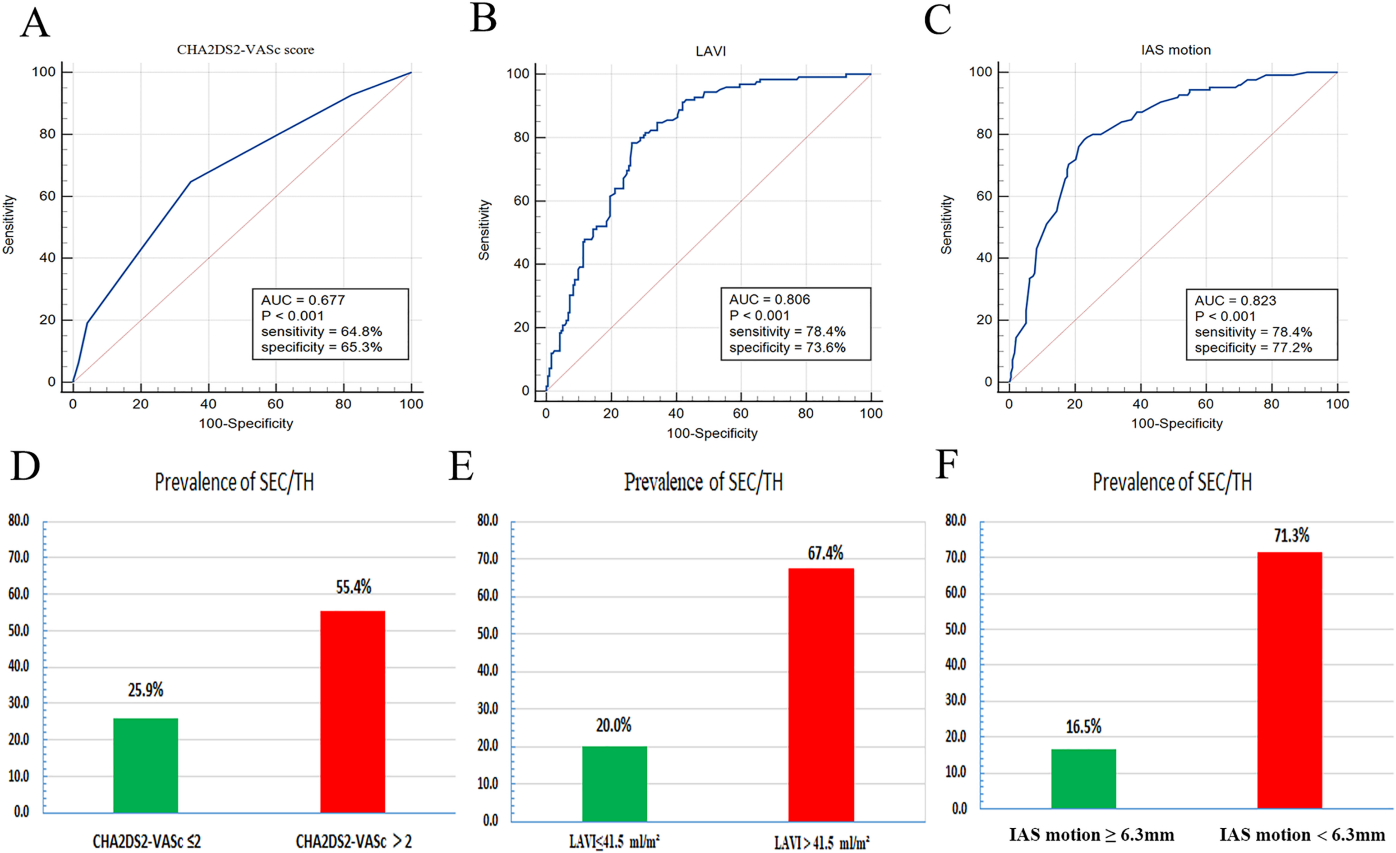
ROC curve analysis **(A–C)** was performed to evaluate the predictive ability of the LA parameters for the detection of spontaneous echo contrast and thrombosis (SEC/TH). Prevalence of SEC/TH stratified by LA parameters derived from ROC analysis **(D–F)**. LA, left atrial; IAS, interatrial septal; LAVI, left atrial volume index; ROC, receiver operating characteristic.

The results of the receiver operating characteristic curve analysis for the prediction of SEC were displayed in Figure 5. Among the tested parameters, IAS motion had the greatest area under the curve (0.823), with a cutoff value of 6.3 mm providing a sensitivity of 78.4% and a specificity of 77.2%. LAVI had the second-highest area under the curve (0.806), with a cutoff value of 41.5 ml/m^2^. Figure 5 also demonstrated the prevalence of SEC/TH stratified by each LA parameter derived from the receiver operating characteristic analysis. When the value of IAS motion was less than 6.3 mm, the incidence of SEC/TH is 71.3% in patients with NVAF.

**Figure 6:**
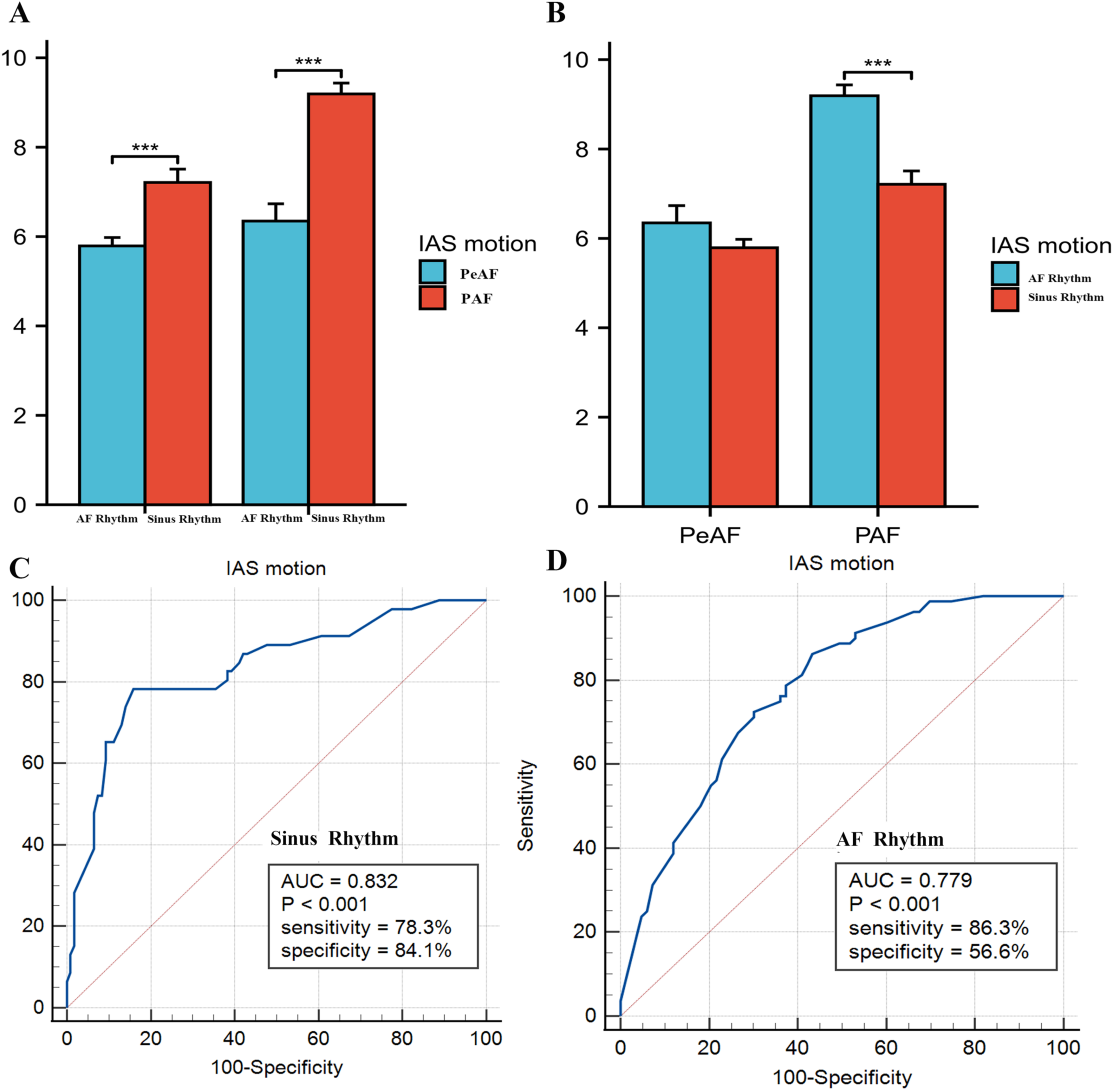
**(A,B)** Compared interatrial septal (IAS) motion between persistent atrial fibrillation (peAF) and paroxysmal atrial fibrillation (PAF) under atrial fibrillation (AF) rhythm and sinus rhythm, respectively. **(C,D)** Showed the ROC curve analysis of IAS motion for predicting risk of SEC/TH in non-valvular atrial fibrillation (NVAF) under sinus rhythm and AF rhythm.

The results showed that there was a difference in IAS septal motion between AF and sinus rhythm in PAF, while no significant difference was found in PeAF, this may be due to the severely impaired left atrial function in patients with persistent atrial fibrillation, resulting in less susceptibility to the effects of arrhythmia. We also analyzed the differences in IAS motion between PeAF and PAF under AF rhythm and sinus rhythm. The results showed that patients with PAF had higher IAS motion than those with PeAF, regardless of whether they were in AF rhythm or sinus rhythm. We further compared the value of IAS motion under sinus rhythm and AF rhythm in predicting thrombosis risk. The results showed that IAS motion under sinus rhythm could better predict thrombus risk, while the predictive efficacy of IAS motion under AF rhythm was slightly attenuated but still had a high AUC value (0.779), The results were shown in Figure 6.

**Figure 7:**
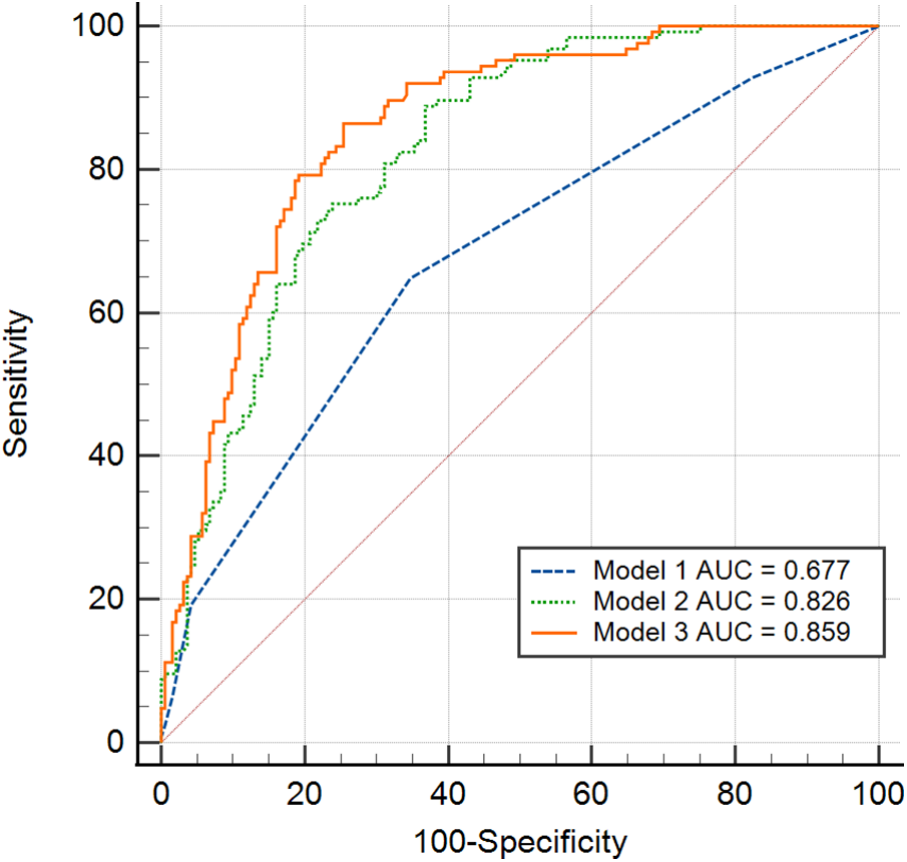
Receiver operating characteristic curve analysis was conducted to compare the various prediction values of SEC/TH. Model 1: CHA_2_DS_2_-VASc scores alone. Model 2: Combined CHA_2_DS_2_-VASc and LAVI with mean E/e’. Model 3: The addition of IAS motion to Model 2. IAS, interatrial septal; LAVI, left atrial volume index; SEC/TH, spontaneous echo contrast and thrombosis.

Figure 7 displayed a comparison of the predicted AUC values for SEC/TH. The second Model that combined CHA_2_DS_2_-VASc, LAVI with mean E/e’ showed superior predictive value compared to CHA_2_DS_2_-VASc scores alone (Model 1) in patients with NVAF (AUC = 0.826 vs. 0.677, *P* < 0.01). Furthermore, the addition of IAS motion to Model 2 enhanced the model's performance, as indicated by Model 3 exhibiting even higher predictive accuracy than Model 2 (AUC = 0.859 vs. 0.826, *P* = 0.02).

## Discussion

In this cross-sectional study of 318 consecutive patients referred for TEE before radiofrequency ablation and/or LAA occlusion, we demonstrate that (1) IAS motion measured by TEE correlates well with LAA flow velocity and LA size and is independently associated with SEC/TH in patients with NVAF. (2) Additionally, lower IAS motion is associated with a higher prevalence of SEC/TH. (3) Furthermore, the integration of IAS motion to CHA_2_DS_2_-VASc, LAVI, and mean E/e’ can provide additional value for the prediction of SEC/TH. We believe that this is the first study to investigate the connection between IAS movement and SEC/TH in patients with NVAF.

The IAS has been linked to various aspects of atrial fibrillation, including its movement, thickness, and potential connection to atrial pressure, volume, and fibrosis (6, 10). Furthermore, its role in the initiation of atrial fibrillation, fat deposition, and atrial conduction times highlights its significance in the pathophysiology of AF. The TEE can offer a more comprehensive understanding of anatomical and functional details. It has been shown that IAS thickness can serve as an indicator of both structural and functional remodeling of LA in patients with AF. A previous study indicated that IAS thickness could potentially serve as an alternative indicator for alterations in the atrial wall's constituents (11). Studies have demonstrated that the IAS motion was primarily influenced by trans-atrial pressure gradient, as well as the contractions of the LA (12). These studies have discovered that during early diastole and atrial contraction, the pressure in the LA is higher than that in the right atrium, resulting in the bending of the septum towards the right. In contrast, during systole, there is a brief moment when the pressure in the right atrium surpasses that of the LA, causing the septum to bend towards the LA. The IAS motion was classified into three patterns based on its shape and movement using TEE in their study. Among these patterns, a fixed curvature pattern of IAS motion was found to be linked with high pulmonary capillary wedge pressure (7). The study by Haji et al. provides valuable insights into the relationship between IAS motion and pulmonary capillary wedge pressure in patients undergoing cardiac surgery. Their findings indicated that a fixed curvature pattern of the IAS motion proved to be the most accurate predictor of increased Pulmonary capillary wedge pressure (PCWP) (13). A recent study proposed that IAS motion might serve as a novel indicator for predicting increased LA pressure (14). However, few studies have explored the correlation between IAS motion and stroke risk in patients with AF.

This study utilizes TEE to quantitative evaluation of the IAS motion and investigate its relationship with SEC/TH. Our findings suggested that IAS motion was independently associated with SEC/TH. Lower IAS motion was linked to a higher incidence of SEC/TH, which had a high sensitivity and specificity. There is a strong connection between high LA pressure and electroanatomic remodeling in patients with AF (15). Previous studies have utilized IAS motion to assess LA pressure in patients with AF and compared it against pressure measured via catheterization. The findings suggest that IAS motion during sinus rhythm could serve as a novel indicator for elevated LA pressure, and lower IAS motion is correlated with higher LA pressure (14). It is still noteworthy that these findings were undetected in NVAF patients during AF rhythm. The E/e’ serves as a significant surrogate marker for LAP (16). This study examines the relationship between IAS motion and mean E/e’ in patients with NVAF. We also found a poor correlation between the IAS motion and mean E/e’ in both PeAF and PAF group. Since our study involves 132 patients (42%) with PAF, and most of the IAS motion measurements were obtained during AF rhythm, which could potentially weaken the association between IAS motion and LA pressure. Our results align with previous research. The relationship between IAS motion and LA pressure may be weakened due to the diminished or decreased atrial contractions during AF.

LAA flow velocity and LA size play crucial roles in the development of SEC and thrombus in patients with AF (17–19). Our study examined the relationship between IAS motion and LAA flow velocity. The results suggest a significant and positive correlation between IAS motion and both filling and emptying velocities in the LAA. LAA flow velocity is an important parameter measured during TEE examination, which can serve as an indicator for thromboembolic risk, especially in patients with AF (20, 21). Due to the irregular and often rapid heart rate in AF, the effective contraction of the atria is compromised, which leads to reduced LAA velocities. Consequently, this can result in blood stasis within the LAA, thereby increasing the risk of thrombus formation. The IAS motion might indirectly reflect conditions that lead to reduced LAA flow velocities because both phenomena could be consequences of atrial dysfunction or pressure imbalances.

Patients with AF often exhibit an enlarged LA, as indicated by an increase in LAVI. This enlargement is typically caused by pressure or volume overload, and is often triggered or worsened by the arrhythmia itself (22). Studies have shown that patients with AF and a larger LAVI have a higher occurrence of thrombus in the LAA and are more likely to suffer from strokes or systemic embolism (19). This study revealed a significant negative correlation between IAS motion and LAVI. The IAS motion can be affected by various factors, such as changes in atrial pressures and volume overload (23). In AF, the disorganized electrical activity leads to the loss of effective atrial contraction. This contributes to an increase in the size and pressures of the LA, which can in turn affect IAS movement due to distorted atrial mechanics and pressure dynamics.

LA remodeling in patients with AF involves alterations in the structure and function due to the arrhythmia. AF leads to the enlargement of the LA, elevated LA pressure and progressive fibrosis (24). These changes can significantly impact stroke risk and affect the prognosis for patients with NVAF. IAS motion, linked to LA pressure, volume, and atrial septal fibrosis in AF patients, is independently associated with SEC/TH in NVAF patients when assessed by TEE. It may serve as a new indicator for stroke risk in NVAF patients. Furthermore, the integration of IAS motion to CHA_2_DS_2_-VASc and commonly used traditional parameters (LAVI and mean E/e’) can provide additional value for the prediction of SEC/TH. The present study effectively captured the link between IAS motion and the occurrence of SEC/TH. Lower IAS motion often occurs in association with SEC/TH. Therefore, understanding this correlation can assist in clinical diagnosis and classifying the risk of thromboembolic events in order to prevent the complications associated with SEC/TH.

This study has some limitations. First of all, this study is a single-center cross-sectional study, and the sample size is limited. It is necessary to conduct a multi-center study with a larger sample size in order to validate the findings. Secondly, there exist some discrepancies in the measurements of IAS motion between sinus rhythm and AF rhythm. Thirdly, the association between IAS motion and LA pressure during AF rhythm was suspicious and it still needed further evaluation. As the mean E/e’ ratio was considered an alternative to LA pressure, it lacks the gold standard for assessing LA pressure through cardiac catheterization. At last, The IAS motion is not suitable for repeated monitoring because TEE is an invasive procedure. Future research will be needed to determine the use of anatomical M-mode technology for assessing IAS motion through TTE.

## Conclusion

Our study utilize TEE to evaluate the motion of the IAS in patients with NVAF and is the first to investigate its correlation with the risk of SEC/TH. we demonstrate that IAS motion measured by TEE correlates well with LAA flow velocity and LA size and is independently associated with SEC/TH in patients with NVAF. Additionally, lower IAS motion is associated with a higher prevalence of SEC/TH. Furthermore, the integration of IAS motion to CHA_2_DS_2_-VASc, LAVI, and mean E/e’ can provide additional value for the prediction of SEC/TH.

## Data Availability

The original contributions presented in the study are included in the article/Supplementary Material, further inquiries can be directed to the corresponding author.

## Supplementary material

Supplementary Figure 1 A comparative analysis of IAS motion within subgroups based on the degree of SEC/TH. IAS, interatrial septal; SEC/TH, spontaneous echo contrast and thrombosis.
