# Supplementary figures and images for: The incremental value of interatrial septum motion in predicting thrombus or spontaneous echo contrast in patients with non-valvular atrial fibrillation: an observational study on transesophageal echocardiography
Source: Front Cardiovasc Med. 2024 Oct 16;11:1366180. doi: 10.3389/fcvm.2024.1366180 (PMC11521842; doi:10.3389/fcvm.2024.1366180)

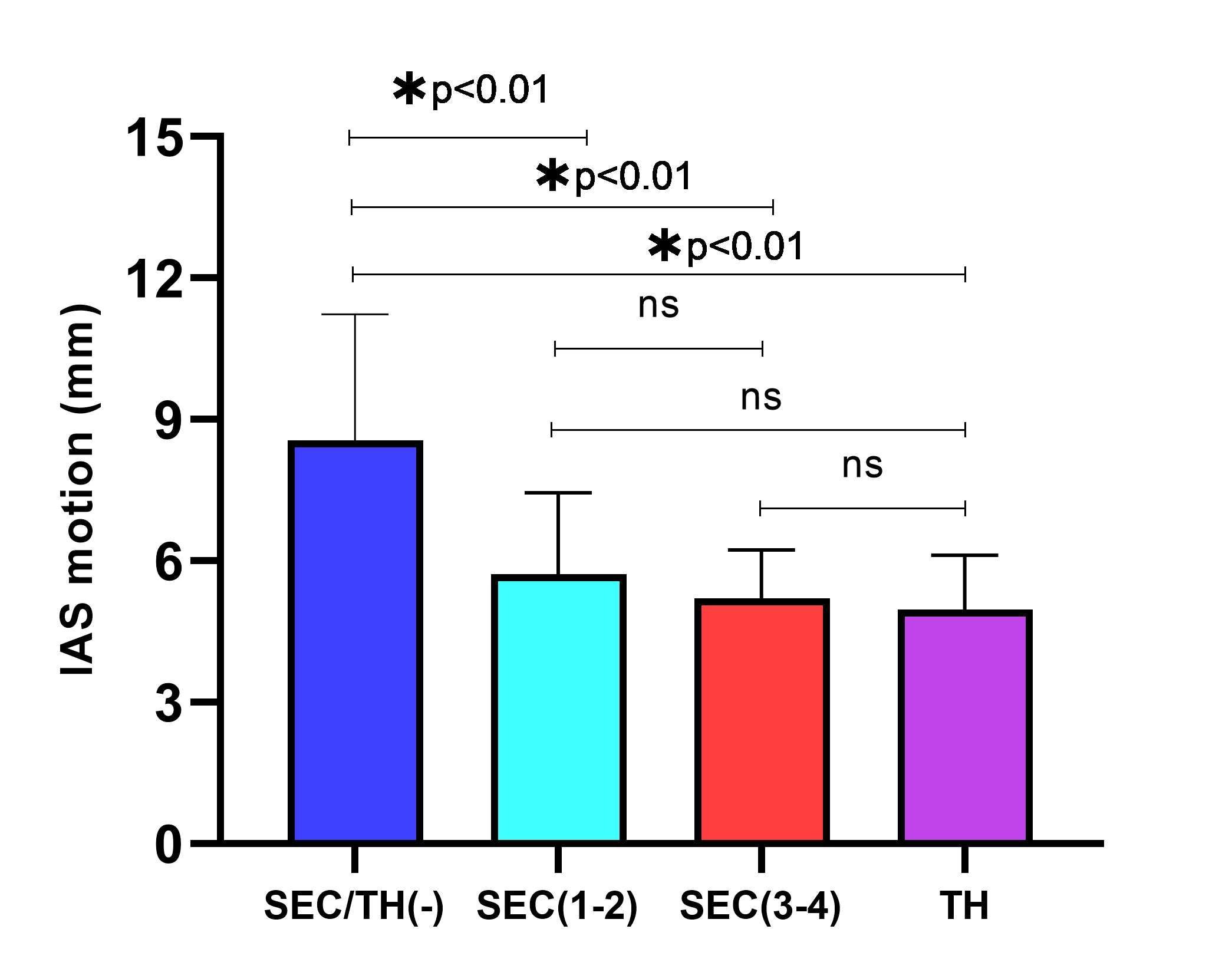

Supplement: Supplementary Figure 1 — A comparative analysis of IAS motion within subgroups based on the degree of SEC/TH. IAS, interatrial septal; SEC/TH, spontaneous echo contrast and thrombosis. [file Image1.tif]
